# The influences of childlessness on the psychological well-being and social network of the oldest old

**DOI:** 10.1186/1471-2318-11-78

**Published:** 2011-11-23

**Authors:** Josefin Vikström, Marie Bladh, Mats Hammar, Jan Marcusson, Ewa Wressle, Gunilla Sydsjö

**Affiliations:** 1 Obstetrics and Gynaecology, Department of Clinical and Experimental Medicine, Faculty of Health Sciences, Linköping University, SE-581 85 Linköping, Sweden; 2 Department of Geriatric Medicine, University Hospital, SE-581 85 Linköping, Sweden; 3 Geriatrics, Department of Clinical and Experimental Medicine, Faculty of Health Sciences, Linköping University, SE-581 85 Linköping, Sweden

## Abstract

**Background:**

The ELSA 85 project is a population-based study with the purpose to learn more about the "elderly elderly". The aim of this part of the ELSA 85 study is to explore the effects of childlessness on the psychological wellbeing, living situation and social support of 85-year old individuals.

**Methods:**

A postal questionnaire was sent to all (650) 85-year old men and women living in Linköping Municipality in 2007. Psychological well-being and social network was measured using a number of questions.

**Results:**

496 individuals participated in the study. No differences in psychological wellbeing were found between the 85-year olds who were childless and those who were parents. The childless 85-year olds were less likely to have relatives close by and to receive help than those who were parents. Individuals of both groups were equally likely to end up in institutional care, to have friends close by and to be in contact with neighbours.

**Conclusions:**

Even though elderly childless individuals have social networks of less support potential than those who are parents there are no differences in certain psychological wellbeing indicators between the two groups. Apparently, childless elderly individuals find ways to cope with whatever negative effects of childlessness they may have experienced.

## Background

According to Lalos [1] and Cousineau and Domar [2], involuntary childlessness can have great psychological consequences for couples who are in their childbearing years and adjusting to the prospect of remaining childless requires the use of psychological coping skills. Meanwhile, voluntarily childless adults often perceive that they are viewed negatively by others and are often asked to explain their choice not to have children [3]. Because of these negative consequences, and the view of children as the major source of support to aging individuals, it is often assumed that childlessness will have negative consequences when childless individuals reach old age [4]. This has in turn led to the belief that the absence of children would leave elderly individuals without support in old age [4].

Several studies have investigated the influence of childlessness on the psychological well-being of middle-aged and elderly individuals using a wide range of outcome measures. In these studies, it was not specified whether the participants were voluntarily or involuntarily childless. A majority of the studies found that being childless does not significantly influence the psychological well-being of elderly individuals [5-8]. Childless middle-aged and elderly individuals do not appear to be lonelier [7], unhappier [9] or more depressed [7-9] and are not less satisfied with their lives [9] than those who are parents. However, contrary to these results, Koropeckyj-Cox [10] found that childless elderly women were both lonelier and more depressed than mothers while no differences between childless elderly men and fathers were found.

The results of some studies indicate that marriage decreases the frequency of occurrence of depressive symptoms [7,8], loneliness [6,7] and increases the satisfaction with life [9] among elderly men and women. Other studies have not found any differences between married and never-married men and women regarding depression [10,11], happiness [11], loneliness [10] or life satisfaction [11,12].

In respect to differences in gender, men are less likely than women to feel depressed [7,11] and lonely [7], but these advantages disappear in the absence of marriage and children [6,7]. In fact, among the elderly individuals who are childless and unmarried, men are more likely than women to feel depressed and lonely [7].

Several studies have also investigated the influence of childlessness on social network and support. In these studies, it was not specified whether or not the childlessness was voluntary or involuntary. The studies showed that childless elderly men and women have smaller social networks [12,13], they are less likely to interact with relatives and more likely to have networks of limited support potential [14] than elderly men and women who are parents. The smaller networks are in part attributable to the lack of children and grandchildren, but there is no evidence that childless individuals have sought to increase social contacts or to extend non-kin networks to compensate for their childlessness [12,13]. However, Keith [6] and Wenger [15] found no differences in social networks and support between childless individuals and parents.

Wenger [15] also found that the elderly who are childless have a greater likelihood than parents of living in institutional care. In concordance with these results, Larsson and Silverstein [16] found in their study of Swedish individuals aged 81 years and older, that while parents receive more informal support, the childless receive more formal support. Thus, it appears that childless men and women are not worse off than parents as a result of any possible weaknesses in their social support networks, even though they do not seem to compensate for their lack of children by increased socialization. They are, however, more likely to end up in institutional care.

To our knowledge, none of the studies on the psychological consequences of childlessness on elderly individuals have focused specifically on the oldest old, the cohort aged 85 years and older, and most studies have excluded the large number of elderly people who live in nursing homes. According to Koropeckyj-Cox [10], when the elderly over the age of 85 are not included in studies, a group in which loneliness and depression would have been more prevalent and devastating is excluded. Many authors [3,7,10] have emphasised the need to explore the influences of childlessness on the oldest old. In fact, according to Grundy and Bowling [17] there is a general lack of data on the circumstances and quality of life of this group of individuals.

Known coping strategies for dealing with involuntary childlessness include helping out with nieces and nephews, remaining in close contact with friends and getting a pet to fill the time that children would have taken up [18]. While younger cohorts of the childless elderly can use coping strategies such as these, the oldest old, and especially those living in nursing homes, may not have this possibility. Both involuntarily and voluntarily childless individuals might find that they, when they reach old age, have to rely on other people for social interaction and instrumental support, something perhaps only close relatives such as a spouse, children or grandchildren are prepared to give.

The objective of this study is to investigate the influences of childlessness, no matter if it is voluntary or involuntary, on the general life satisfaction, sense of meaning in life, happiness, depression and loneliness in a population of 85-year old individuals, and to examine the possible interactions with marital status and gender. The second objective is to investigate how childlessness influences social support network and the probability of ending up in institutional care among the 85-year old men and women.

Our first hypothesis is that childless men and women will experience a lower sense of psychological wellbeing than parents and that being unmarried, widowed or being a woman will increase feelings of depression and loneliness and decrease happiness among those who are childless. Our second hypothesis is that the elderly who are childless will have social networks of less support potential (the support potential of the social networks is indicated by the likelihood of receiving help) and be more likely to end up in institutional care than the elderly who are parents. Our third hypothesis is that the childless 85-year old men and women living in institutional care will score lower on self-rated health and higher on sense of loneliness and depression than those who are childless and are living out in the community since those who are living in institutional care will be frailer and more dependent on others for social interaction and support.

## Methods

The analyses are based on data from the Elderly in Linköping Screening Assessment, ELSA 85, a population study of 85-year olds living in Linköping, Sweden. The purpose of the ELSA 85 study is to learn more about the population known as the "elderly elderly". The "elderly elderly" often suffer from multiple chronic illnesses and require large amounts of health care resources. For further information about the purpose and background of the ELSA 85 study we refer to a recently published article on the topic [19].

### Participants

A postal questionnaire was sent to all individuals born in 1922 who lived in Linköping municipality in 2007. Linköping is a university city in southeast Sweden with a municipality consisting of mainly urban inhabitants but also some rural areas. In 2007, Linköping municipality had 140367 inhabitants [20]. The names and addresses of the eligible participants were obtained from the Swedish population register. Included in the postal questionnaire was information about the nature of the study, that all data were to be treated confidentially and options for participation in each phase of the study or the choice not to participate at all. A self-addressed envelope was enclosed for the written consent, which was a requirement for participation in the study.

### Procedure

The questionnaire contained questions about demographics, living situation and social network, use of mobility service, health care, home care service and assistive technology. Included were also questions about loneliness, medical problems and medication. The majority of the questions were to be answered by fixed choice questions; the exceptions were questions about profession, medications, illnesses, reasons for need of home care service and medical care and finally, worries about the future, which were investigated using open questions. Also enclosed was EuroQol- 5D (EQ-5D**) **[21] , an instrument used for assessing health-related quality of life, with an included visual analogue scale recording self-rated health ranging from 0, which is the worst imaginable health state, to 100, which is the best imaginable health state [22].

The dependent variables were measured using a series of questions. The response alternatives for "number of children" ranged between 0 "No children" to 4 "Four or more children". Response alternatives 1 through 4 were subsequently grouped together under the subheading "children" while response alternative 0 was renamed "no children". The response alternatives for "marital status" in the postal questionnaire was 1 "Married/cohabiting", 2 "Unmarried" and 3 "Widow/widower". Gender was measured using the alternatives 1 "Woman" and 2 "Man".

Psychological well-being was measured using questions about loneliness, general life satisfaction, sense of meaning in life, happiness and depressive symptoms. These were the questions included in the ELSA85 project that contribute to the general measure psychological well-being and that had been used as measures in other studies on the consequences of childlessness on psychological well-being among the elderly. While questions about loneliness were included in the postal questionnaire, the remainder of the questions regarding psychological well-being was posed by the occupational therapist during the interview in the participant's home.

The prevalence of loneliness was measured using the answer to a single question: "Do you sometimes feel lonely?" with response alternatives 1 "Yes, often", 2 "Yes, sometimes", 3 "No, rarely", 4 "No, never" and 5 "No, I wished that I had more time to myself". For the logistic regression analyses the response alternatives were dichotomised into "Lonely" consisting of response alternatives 1-3 and "Not lonely" consisting of response alternatives 4-5. The strength of feelings of loneliness among those who chose response alternative 1 or 2 to the prevalence question was measured by the answer to the question: "How strong is your feeling of loneliness?", with response alternatives ranging from 1 "Very strong" to 5 "Very weak".

The focus of the interview performed by the occupational therapist in the participant's home was activities of daily living, fall risk, participation and autonomy, perceived problems in occupational performance and interests.

Depression was screened for using the Geriatric Depression Scale -20 (GDS -20), which is a questionnaire with 20 questions [23]. Five of the questions were excluded to form GDS-15, which is considered a valid and reliable scale for screening for depression in the elderly [24]. The responses were recorded and added to create a total depression score with a possible range from 0 to 15. The cut-off value was set at ≥ 5, which has a good sensitivity and specificity when screening for depression among the elderly [25]. The values for depressive symptoms were thus 1 "Depressed", when the GDS-15 score was ≥ 5, and 2 "Not depressed" when the GDS-15 score was < 5. Questions about general life satisfaction, happiness and meaningfulness were also included in the interview.

General life satisfaction was measured by the answer to a single, global question: "What do you think of your life in general at the moment?", with response alternatives ranging from 1 "Very good" to 5 "Very bad". Also measured was the sense of meaning in life. The single question: "Do you think that your life is meaningful at the moment?" was used with response alternatives ranging from 1 "To the highest degree meaningful" to 5 "without meaning". Self-rated happiness was measured with the single global question: "Do you feel happy at the moment?" with response alternatives 1 "To the highest degree happy", 2 "Quite happy", 3 " Neither happy nor unhappy", 4 "Quite unhappy and 5 "Very unhappy". For the logistic regression analyses the response alternatives were dichotomised into "Happy" consisting of response alternatives 1-3 and "Unhappy" consisting of response alternatives 4-5. The participants were also asked to respond to the question "In general, would you say that your health is" with the response alternatives ranging from 1 "Excellent" to 5 "Bad".

The nature of the individual's social network was examined by posing questions about: "social network, relatives", "social network, friends" and "are you in contact with your neighbours?". There were three response alternatives for "social network, relatives": 1 "I have got relatives close by", 2 "I only have relatives at a different location", 3 "I do not have any relatives". There were two response alternatives for "social network, friends": 1 " I have friends close by", 2 " I do not have friends close by". There were three response alternatives for the question "are you in contact with your neighbours?": 1 "I am in close contact with my neighbours", 2 " I am in some contact with my neighbours", 3 "I do not have any contact with my neighbours". The strength of the social networks was examined through the question "Do you receive help from family or friends?" with response alternatives: 1 "No", 2 "Yes, from husband/wife/cohabiter", 3 "Yes, from a sibling", 4 "Yes, from children/grandchildren" and 5 "Yes, from a friend or friends".

### Statistical analyses

Statistical analyses were performed using SPSS 16.0 (SPSS, Chicago, USA). Chi-square tests were used with the purpose to find significant differences in the distribution of variables across the parental status groups. Chi-square tests were also performed on the differences in three dependent variables (loneliness, depression and self-rated health) across the two parental status groups subdivided by living situation (living out in the community or in a nursing home). For these analyses, loneliness, depression and self-rated health were defined as binary variables taking on the values of 1 (lonely/depressed/not in good health) and 0 (not lonely/not depressed/in good health). When the sample sizes fell below n = 5 per cell and p ≤ 0,05, Fisher's exact tests were performed manually to further test for significance.

Multivariate logistic regression analyses were performed in order to further investigate the influences of parental status on depression, loneliness and happiness while controlling for gender, marital status, living situation and self-rated health, factors that are known to influence the psychological well-being among the elderly. For these analyses depression, loneliness and happiness were recoded as binary and modelled separately as dependent variables with parental and marital status, health, gender and living situation as independent predictors. They were entered as; parental status with levels parent (reference level) and childless, marital status with levels married (reference level), unmarried and widowed, gender where woman was reference, housing with levels housing in the community (reference level), residential care and nursing home and health status with levels good (reference level) and bad health. Follow-up bivariate logistic regression analyses, including parental status and one control variable each time, were performed as well as a correlation analyses.

### Ethics

If the individual was unable to answer the questionnaire by him- or herself or to decide whether or not to participate in the study, a relative or a caretaker could provide help with filling out the questionnaire. Any medical problems discovered during the visit at the Department of Geriatric medicine were forwarded to a general physician in primary care or another clinic for further evaluation. The study was approved by the Regional Ethics Board in Linköping (*§*141-06).

## Results

**Figure 1:**
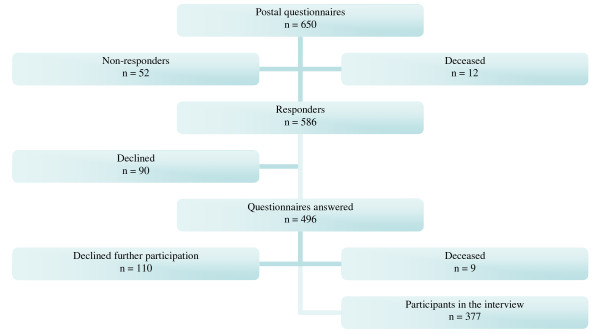
**Flow chart of the ELSA 85 study**.

There were 650 85-year olds living in Linköping municipality. As it is shown in Figure 1, the first part of the study consisted of a postal questionnaire, which was sent out to all 650 85-year olds. Out of these, 90 percent (n = 586) replied to the offer of participation in the first part of the study. Those who did not reply consisted of 52 individuals who could not be reached, either by post or by telephone, and 12 individuals who had died. While 90 individuals declined, written consent was received from 76 percent (n = 496) of the 85-year olds, all of whom had answered the postal questionnaire. In the second part of the study an occupational therapist contacted all participating 85-yearolds by phone to schedule an interview performed by the occupational therapist in the participant's home. Out of the 496 individuals, 76% (n = 377) were positive to further participation in the study. Among those who did not participate further in the study, 110 individuals had declined further participation and 9 individuals had died.

There were no statistically significant differences in gender between those who filled out the questionnaire, and thus chose to participate in the study, and those who chose not to participate, but there was a difference between the groups concerning housing (p < .05). Those who consented to participate in the study were more likely to live in a house or apartment, while those who did not reply or chose not to participate were more likely to live in institutional care.

As shown in Table 1 the majority of the 85-year old individuals studied were women, most of the participants were married or widowed and a clear majority were parents. There was a statistically significant difference in the distribution of marital status between men and women. While the majority of the men, 67.5%, were married, the majority of the women, 59.2%, were widowed. The difference in the number of unmarried individuals was smaller, 12.0% of the women and 7.9% of the men were unmarried. Elementary school was the highest level of education that the majority of the respondents had completed (see Table 1).

**Table 1:** Sample characteristics by parental status group.

	Total		Parents		Childless		p**
	n	%*	n	%*	n	%*	
**Independent variables**							
**Gender**							
n	495		438		57		
Man	187	38.1	164	37.4	23	40.4	
Woman	308	62.2	274	62.6	34	59.6	
							.670
**Marital status**							
n	494		437		57		
Married	214	43.3	200	45.8	14	24.6	
Unmarried	52	10.5	31	7.1	21	36.8	
Widowed	228	46.2	206	47.1	22	38.6	
							.000 ^+^
**Education**							
n	490		433		57		
Elementary school	344	70.2	309	71.4	35	61.4	
Secondary grammar school	104	21.2	88	20.3	16	28.1	
College/University	42	8.6	36	8.3	6	10.5	
							.298

* Some percentages do not add up to 100% because of rounding.

** Calculated with a Pearson Chi-square test. Significance level at ≤ .05.

^+ ^Statistically significant.

There were no differences regarding the distribution of men and women or highest level of education across the parental status groups (see Table 1). However, a difference in marital status was found. The men and women of the parental group were more likely to be married than were the men and women of the childless group, and they were also slightly more likely to be widowed (see Table 1).

The majority of the 85-year old men and women sometimes, rarely or never felt lonely and, among those who did experience some feelings of loneliness, the feelings were neither strong nor weak (see Table 2). No significant differences were found between the childless group and the parental group in regards to the prevalence and strength of feelings of loneliness. For the logistic regression analyses the outcome measures were dichotomised into "Lonely" (n = 194) and "Not lonely" (n = 295). These analyses showed that men were lonelier than women and that those who were widowed were less likely to feel lonely compared to those who were married (see Table 3). It was also shown that self-rated health and living situation influenced feelings of loneliness. The 85-year olds with a poor self-rated health were less likely to feel lonely compared to those with a good self-rated health and those who were living in a nursing home were less likely to feel lonely than those who were living out in the community (see Table 3). The bivariate logistic regression analyses showed that those living in residential care were also less likely to feel lonely than those living out in the community. However, these differences disappeared when controlling for other factors in the multivariate logistic regression analyses.

**Table 2:** The relationship between parental status and psychological well-being: Distribution and significance testing.

	Total		Parents		Childless		p**
	n	%*	n	%*	n	%*	
**Dependent variables**							
**Rate of feelings of loneliness**							
n	487		430		57		
Often	40	8.2	33	7.7	7	12.3	
Sometimes	154	31.6	136	31.6	18	31.6	
Rarely	150	30.8	132	30.7	18	31.6	
Never	141	29.0	127	29.5	14	24.6	
Would like more time alone	2	.4	2	.5	0	0	
							.734
**Strength of feelings of loneliness**							
n	266		230		36		
Very strong	9	3.4	7	3.0	2	5.6	
Quite strong	43	16.2	38	16.5	5	13.9	
Neither strong nor weak	117	44.0	100	43.5	17	47.2	
Quite weak	57	21.4	51	22.2	6	16.7	
Very weak	40	15.0	34	14.8	6	16.7	
							.857
**Depression. GDS-15**							
n	494		437		57		
Score ≤ 4p	331	67.0	294	67.3	37	64.9	
Score ≥ 5p	163	33.0	143	32.7	20	35.1	
							.721
**Life satisfaction**							
n	348		310		38		
Very good	143	41.1	132	42.6	11	28.9	
Quite good	162	46.6	142	45.8	20	52.6	
Neither good nor bad	26	7.5	21	6.8	5	13.2	
Quite bad	13	3.7	12	3.9	1	2.6	
Very bad	4	1.1	3	1.0	1	2.6	
							.323
**Sense of meaning in life**							
n	346		308		38		
To the highest degree	113	32.7	105	34.1	8	21.1	
Quite meaningful	178	51.4	158	51.3	20	52.6	
Neither meaningful nor meaningless	26	7.5	21	6.8	5	13.2	
Quite meaningless	26	7.5	22	7.1	4	10.5	
Without meaning	3	.9	2	.6	1	2.6	
							.229
**Happiness**							
n	346		308		38		
To the highest degree happy	101	29.2	95	30.8	6	15.8	
Quite happy	169	48.8	150	48.7	19	50.0	
Neither happy nor unhappy	49	14.2	43	14.0	6	15.8	
Quite unhappy	25	7.2	19	6.2	6	15.8	
Very unhappy	2	.6	1	0.3	1	2.6	
							.038^++^

* Some percentages do not add up to 100% because of rounding.

** Calculated with Pearson Chi Square. Significance level at ≤.05.

^+ ^Statistically significant.

^++^Due to sample size ≤5 and p ≤ .05 Fisher's exact test was performed with p ≤ .05.

**Table 3:** Effects of parental status on loneliness, depression and self-rated health: Multivariate logistic regression analyses

	Loneliness			Depression			Happiness		
	OR	p	**C.I**.	OR	p	**C.I**.	OR	p	**C.I**.
**Variable**									
Independent variable									
**Parental status**									
Childless (ref)	Reference level			.Reference level			.Reference level		
Parent	1.271	.563	.563-2.868	1.645	.466	.431-6.279	.699	.536	.211-2.244
									
Control variables									
Marital status									
Married (ref)	Reference level			Reference level			Reference level		
Unmarried	.502	.133	.204-1.235	1.457	.576	.389-5.453	4.430	.031*	1.145-17.144
Widowed	.244	.000***	.141-.423	1.079	.861	.463-2.512	1.885	.249	.642-5.536
									
Gender									
Woman (ref)	Reference level			Reference level			Reference level		
Man	1.870	.020*	1.106-3.164	.797	.596	.345-1.842	1.508	.403	.576-3.944
									
Housing									
Housing in the community (ref)	Reference level			Reference level			Reference level		
Residential care	1.093	.122	.398-3.003	1.374	.026*	.399-4.731	2.525	.239	.730-8.720
Nursing home	.163	.041*	.029-.932	8.219	.007**	1.767-38.229	2.699	.143	.408-17.844
									
Health status									
Good health (ref)	Reference level			Reference level			Reference level		
Bad health	.522	.013**	.313-.870	4.774	.000**	2.223-10.254	4.511	.001***	1.810-11.245
									
Constant	0.32	.000		2.730	.046		.023	.000	

OR = Odds Ratio

*p < .05. **p < .01. *** p < .001.

C.I.: 95% for OR

The satisfaction with life among the participants was generally very or quite good and a majority of the respondents thought that their lives had meaning (see Table 2). No differences were found between the childless group and the parental group regarding satisfaction with, or meaning in, life. The level of happiness among 85-year old men and women was generally high. Most respondents reported that they were happy to the highest degree or were quite happy. Interestingly, those in the parental group were significantly happier than those in the childless group (see Table 2). The outcome measures for the logistic regression analyses were dichotomised into "Happy" (n = 319) and "Unhappy" (n = 27). These analyses showed that, when other factors were controlled for, the differences in happiness across the parental status groups disappeared (see Table 3). However, the multivariate and bivariate logistic regression analyses showed that those in poor health were significantly happier than those in good health and that the unmarried were more likely than the married to be happy while there were no differences between the married and the widowed. In regards to housing, those living in residential care were, in the bivariate logistic regression analyses, found to be happier than those who were living out in the community. However, these differences disappeared in the multivariate logistic regression analyses.

As shown in Table 2, 33% of the participants scored above the cut off value (≥ 5) on the GDS-15 scale and were thus defined as being depressed (see Table 2). The number of participants with scores ≥ 5 was 161, the number scoring ≤ 4p was 331. While there were no differences in depression depending on parental- or marital status groups, living in institutional care and experiencing a poorer self-rated health increased the likelihood of feeling depressed (see Table 3). The bivariate logistic regression analyses showed that women were significantly more likely than men to feel depressed. This difference disappeared when controlling for other factors in the multivariate logistic regression analyses. Those living in nursing homes were 8 times as likely to feel depressed than were those living out in the community and those with bad health were 4.5 times as likely as those in good health to experience depression. The difference between those living out in the community and those living in residential care was not found in the bivariate logistic regression analyses showing that this difference was a result of an interaction with other confounders. However, confidence intervals for the residence variables in the regression analyses were very large and should be interpreted with caution.

We also performed a correlation analyses which showed a low coliniality between all dependent variables except for gender and marital status.

The parental group and the childless group were subdivided by the individuals' living situation, meaning living out in the community or in institutional care. Significant differences between the four subgroups were found in regards to depression, loneliness and self-rated health. Those who lived in institutional care were more likely to be depressed and have a poorer self-rated health but these differences were not affected by parental status. The analyses also indicated that those living in institutional care were lonelier than those living out in the community, regardless of parental status. However, while controlling for other factors in the multivariate logistic regression analyses (table 3) it was shown that those living in nursing homes were less likely to be lonely than those living out in the community, results which were repeated in the bivariate logistic regression analyses.

As shown in Table 4 the majority of the participants lived in out in the community and there were no statistically significant differences in living situation or self-rated health between those who were childless and those who were parents.

**Table 4:** The relationship between parental status and self-rated health, living situation and social network: Distribution and significance testing.

	Total		Parents		Childless		p**
	n	%*	n	%*	n	%*	
**Dependent variables**							
**Self-rated health**							
n	345		309		36		
Excellent	16	4.6	14	4.5	2	5.6	
Very good	46	13.3	41	13.3	5	13.9	
Good	167	48.4	153	49.5	14	38.9	
Ok	109	31.6	96	31.1	13	36.1	
Bad	7	2.0	5	1.6	2	5.6	
							.469
**Living situation**							
n	493		436		57		
Living in the community	438	88.8	389	89.2	49	86.0	
Residential care	30	6.1	26	6.0	4	7.0	
Nursing home	25	5.1	21	4.8	4	7.0	
							.728
**Social network**							
**Relatives**							
n	489		433		56		
Close by	409	83.6	374	86.4	35	62.5	
At a different location	78	16.0	59	13.6	19	33.9	
No relatives	2	0.4	0	0	2	3.6	
							.000^++^
**Friends**							
n	479		423		56		
Close by	424	88.5	373	88.2	51	91.1	
Not close by	55	11.5	50	11.8	5	8.9	
							.524
**Neighbours**							
n	480		424		56		
Close contact	142	29.6	126	29.7	16	28.6	
Some contact	272	56.7	240	56.6	32	57.1	
No contact	66	13.8	58	13.7	8	14.3	
							.981
**Received help**							
n	475		422		53		
No help	139	29.4	112	26.5	27	50.9	
From spouse	117	24,6	108	25,6	9	17.0	
From siblings	14	2,9	6	1,4	8	15.1	
From children/grandchildren	184	38,7	183	43,4	1	1,9	
From friends	21	4,4	13	3,1	8	15,1	
							.000^++^

* Some percentages do not add up to 100% because of rounding.

** Calculated with Pearson Chi Square. Significance level at p ≤ 0.05

^+ ^Statistically significant.

^++^Due to sample size ≤5 and p ≤ .05 Fisher's exact test was performed with p ≤ .05.

It appears that the social networks of the 85-year old men and women were functional. Most 85-year old individuals had relatives and friends close by (see Table 4) and the majority were in some contact with their neighbours. No differences across parental status groups were found in regards to having friends close by or being in contact with neighbours. A larger percentage of those who were parents reported having relatives close by than did those who were childless. The childless men and women were also less likely to receive help. Among the 70.6% of the 85-year old individuals who received help, those in both groups were most likely to be given help from relatives, but those who were childless were more likely to receive help from friends than those who were parents. It was most common for the 85-year old parents to receive help from their children or grandchildren while the childless individuals most often received help from their spouses (see Table 4).

## Discussion

Contrary to the hypotheses posed at the beginning of this study, the results are consistent with those of previous studies [4-7]; childless individuals do not score differently than parents on different measures of psychological well-being. Those who are childless are not lonelier than parents and the feelings of loneliness that they do experience are not stronger than those of parents. They are not more depressed or less satisfied with their lives than parents are, they do not experience that their lives have less meaning than do parents and they are not unhappier than parents, once other factors are controlled for (see Table 3). Gender only influences feelings of loneliness, not depression or happiness while marital status influenced both loneliness and happiness. Our results are contrary to those of Zhang and Hayward [6] and Keith [5]; in showing that the widowed are less likely to feel lonely than the married. We also found that the unmarried are happier than the married. These surprising results persisted in the bivariate logistic regression analyses showing that they were not a result of their interaction with other confounders. We also found that men are more prone to loneliness than women. Earlier studies [6,7] have shown that men are less likely than women to feel alone but that these differences disappear in the absence of marriage and children. Since the majority of the men included in our study were married our results are surprising.

We had hypothesised that the 85-year old men and women who were childless would have social support networks of less support potential and would have a greater likelihood than the 85-year old parents of ending up living in institutional care. In accordance with this hypothesis, those of the childless group did have social support networks of less support potential than those in the parental group. This was indicated by the parents' greater likelihood of receiving help. These results are not surprising since the childless men and women were less likely to be married than were the parents and since children or grandchildren were most likely to provide their elderly parents with help. However, contrary to our hypothesis, we found that childless men and women were not more likely than parents to end up living in institutional care. It is possible that there will be a difference as the health of the 85-year old med and women deteriorates.

We had also hypothesised that those of the childless group who were living in nursing homes would feel lonelier, more depressed and have a poorer self-rated health, than those of the childless group who lived out in the community. We formulated this hypothesis because we thought that the childless men and women, as a result of their advanced age, would not be able to make up for not having had children by employing various measures of coping such as going out to see friends, keeping a pet or helping out with nieces and nephews. Our hypothesis was partially contradicted. The chi-square tests showed that the group of parents and the group of childless individuals living in institutional care were more likely to feel depressed, lonely and had a poorer self-rated health than the group of parents and the group of childless individuals who lived out in the community. For these analyses the levels of housing were divided into two categories: living out in the community or in institutional care. For the logistic regression analyses the levels of housing were divided into three categories: living out in the community, residential care or nursing home. These analyses showed that those living in nursing homes had an increased risk of depression compared to those living out in the community while the differences in the prevalence of loneliness were actually conflicting in the chi-square tests and the logistic regression analyses. However, given that the chi-square tests combining living situation and parental status resulted in small sample sizes and the multivariate logistic regression analyses also controlled for other factors, the logistic regression analyses should be considered more trustworthy. These results also persisted in the bivariate logistic regression analyses. Since living in a nursing home means being constantly surrounded by other patients and health-care personnel, it seems reasonable that these individuals were less prone to feel lonely. It is not surprising that individuals living in nursing homes have a poorer self-rated health and are more prone to depression than are those living out in the community.

Health status also influenced the tendency towards feeling lonely, depressed and happy. As can be expected, being in bad health increased the chances of feeling depressed. However, in both the multivariate and the bivariate logistic regression analyses it was found that those who were in bad health were more likely to feel happy and less likely to feel lonely. Could it be that those in bad health are better at appreciating life and therefore are happier? In regards to loneliness, the explanation might be that those who are in bad health are more likely to live in nursing homes and spend more time at hospitals and thus are less likely to feel lonely.

Regarding the differences in social network, those in the childless group were less likely to have relatives close by than were those in the parental group, but no differences between the groups were found in regards to contact with friends and neighbours. These results are consistent with those of other studies which have found that childless men and women are less likely than parents to interact with relatives [5,13], that they have networks of limited support potential [13], but that they do not compensate for their lack of contact with relatives by extended non-kin networks [5,11,12]. However, a definite conclusion about the differences in the social networks between the childless group and the parental group cannot be drawn given that the questionnaire did not contain questions about the number of friends or relatives present and thereby not about the size of the social networks.

In summary, our results show no differences in psychological well-being between the childless elderly and the parental elderly even though the childless individuals generally have weaker social support systems and are likely to have suffered from negative effects of their childlessness on their well-being earlier in life [1,2]. We therefore ask, what might be the reason for this seeming paradox?

First, it must be considered that the results could have turned out differently if alternative methods, such as qualitative interviews, had been employed. Previous studies [3,18] using such methods did find that childlessness influences the wellbeing of elderly individuals negatively.

The lack of negative effects of childlessness on the wellbeing of elderly individuals might also be a result of the ability of these individuals to cope. In this study it was hypothesised that the coping skills that are successfully used by the younger elderly would become even less accessible to those who are "elderly elderly" and frailer and thus that these individuals would suffer more from not having children around for support and help. However, even though the participants of this study were older than those of previous studies, the results show that the majority lived in out in the community and seemed to be feeling well psychologically. It is therefore possible that the participants were capable of using coping strategies such as spending time with a neighbour, enjoying the company of a dog or going out for a daily walk with a friend, factors which might be more important for the well-being of an elderly individual than having children who may not have the time to visit their parents or with whom the parents perhaps are not close.

In a western country like Sweden with a good social security system where there are no traditions that involve several generations living together it is not surprising that parental status does not influence the tendency of elderly people having to move into institutional care nor their psychological wellbeing once they reside in institutional care. However, it is possible that those who were childless to a greater extent were dependent on formal care, for example the use of home care service, even if they were not more likely to end up living in institutional care. Also, our results show that there were advantages to living in institutional care as these individuals were less lonely than those living out in the community.

A major limitation of this study is that it is cross-sectional, which means that no conclusions about causality can be drawn. It was not specified whether or not stepchildren, adopted children or children that had died were to be counted as children and no question was asked to determine if the childlessness was voluntary or involuntary. Thus it is possible that individuals in the childless group had previously had children which might influence their psychological wellbeing in both a negative and a positive direction. Having experienced the death of a child might have influenced psychological wellbeing in a negative direction while it is possible that these individuals had grandchildren for help and support. Being voluntarily or involuntarily childless might not influence social network and support in old age and might also influence psychological wellbeing among the childless in a negative or a positive direction. For some individuals loneliness might be easier to handle knowing that the childlessness was the result of fate while other individuals might more easily be able to handle being lonely knowing that they were childless by choice. The quality of the relationship between the parents and their children was not examined. Naturally this might have influenced the results. Having a poor relationship with one's children might influence psychological wellbeing as much as not having had children and also influence the tendency of the children to provide their parents with help and support. There was a lack of information about the distribution of parental and marital statuses among the non-participants and thus about possible selection of different parental or marital status groups into the study. The fact that the 85-year olds living in institutional care were less likely to participate in the study might have influenced the results. It is probable that the most vulnerable elderly are likely to have died or become institutionalised before reaching the age of 85, and would therefore not be adequately represented as participants of this study. This possible selection of healthier elderly individuals might have resulted in an overly positive image of the psychological wellbeing of elderly individuals. Another major limitation is the small number of childless individuals, resulting in a lack of power, which might have contributed to the lack of significant differences found between the parents and the childless individuals in regards to psychological wellbeing. Also, the use of single-item scales might have influenced the results. For example, loneliness and happiness was based solely on the participants subjective assessments.

## Conclusions

More research is required to learn about the implications of childlessness on the psychological well-being of "elderly elderly" individuals. No previous study has specifically focused on an age cohort as old as 85 years and how parental status influences their psychological wellbeing and social network. Our findings indicate that even though there are differences in the support potential and constitution of the social support networks across the parental status groups, there are no differences in psychological wellbeing between the elderly childless and parents. This is possibly a result of the fact that the majority of the participants lived out in the community and were in good health and that these two conditions help them to retain their ability to cope with whatever negative effects of childlessness they may have experienced. Thus, it appears that having children or remaining childless does not generally influence psychological wellbeing in old age.

## Competing interests

The authors declare that they have no competing interests.

## Authors' contributions

JV drafted the manuscript and participated in the analyses and interpretation of data, MB performed the statistical analyses, MH conceived of the study, participated in its design and interpretation of data, JM and EW conceived of the study, and participated in its design, coordination and interpretation of data, GS conceived of the study, participated in its design, interpretation of data and helped draft the manuscript. All authors have read and approved the final manuscript.

## Pre-publication history

The pre-publication history for this paper can be accessed here:

http://www.biomedcentral.com/1471-2318/11/78/prepub

## References

[B1] Lalos A Breaking bad news concerning fertility Hum Reprod 1999 14 581 585 10.1093/humrep/14.3.581 10221678

[B2] Cousineau T Domar A Psychological impact of infertility Best Pract Res Clin Obstet Gynaecol 2007 29 293 308 10.1016/j.bpobgyn.2006.12.003 17241818

[B3] Jeffries S Konnert C Regret and psychological well-being among voluntarily and involuntarily childless women and mothers Int J Ageing Hum Dev 2002 54 89 106 10.2190/J08N-VBVG-6PXM-0TTN 12054274

[B4] Alexander B Rubinstein R Goodman M Luborsky M A path not taken: a cultural analysis of regrets and childlessness in the lives of older women Gerontologist 1992 32 618 626 10.1093/geront/32.5.618 1427273

[B5] Glenn N McLanhan S The effects of offspring on the psychological well-being of older adults J Marriage Fam 1981 43 409 421 10.2307/351391

[B6] Keith P A comparison of the resources of parents and childless men and women in very old age Fam Relat 1983 32 403 409 10.2307/584618

[B7] Zhang Z Hayward M Childlessness and the psychological well-being of older persons J Gerontol B Psychol Sci Soc Sci 2001 56 S311 S320 10.1093/geronb/56.5.S311 11522813

[B8] Bures R Koropeckyj-Cox T Loree M Childlessness, parenthood, and depressive symptoms among middle-aged and older adults J Fam Issues 2009 30 670 688 10.1177/0192513X08331114

[B9] Koropeckyj-Cox T Pienta A Brown T Women of the 1950s and the "normative" life course: the implications of childlessness, fertility timing and marital status for psychological well-being in late midlife Int J Aging Hum Dev 2007 64 299 330 10.2190/8PTL-P745-58U1-3330 17703677

[B10] Koropeckyj-Cox T Loneliness and depression in middle and old age: are the childless more vulnerable? J Gerontol B Psychol Sci Soc Sci 1998 53 S303 S312 9826972 10.1093/geronb/53b.6.s303

[B11] Connidis I McMullin J To have or have not: parent status and the subjective well-being of older men and women Gerontologist 1993 33 630 636 10.1093/geront/33.5.630 8225007

[B12] Dykstra P Wagner M Pathways to childlessness and late-life outcomes J Fam Issues 2007 28 1487 1518 10.1177/0192513X07303879

[B13] Dykstra P Off the beaten track: childlessness and social integration in late life Res Aging 2006 28 749 767 10.1177/0164027506291745

[B14] Dykstra P Hagestad G Childlessness and parenthood in two centuries: different roads different maps? J Fam Issues 2007 28 1518 1533 10.1177/0192513X07303881

[B15] Wenger C Ageing without children: rural Wales J Cross Cult Gerontol 2001 16 79 109 10.1023/A:1010699231743 14617994

[B16] Larsson K Silverstein M The effects of marital and parental status on informal support and service utilization: a study of older Swedes living alone J Aging Stud 2004 18 231 244 10.1016/j.jaging.2004.01.001

[B17] Grundy E Bowling A Enhancing the quality of extended life years. Identification of the oldest old with a very good and very poor quality of life Aging Ment Health 1999 3 199 212 10.1080/13607869956154

[B18] Wirtberg I Möller A Hogström L Tronstad S Lalos A Life 20 years after unsuccessful infertility treatment Hum Reprod 2007 22 598 604 17124258 10.1093/humrep/del401

[B19] Nagga K Dong H Marcusson J Skoglund S Wressle E Health-related factors associated with hospitalization for old people: Comparisons of elderly aged 85 in a population cohort study Arch Gerontol Geriatr in press 10.1016/j.archger.2011.04.023 21640394

[B20] Scb.se [Internet] Stockholm: Statistiska centralbyrån http://www.scb.se [updated 2009 Feb 17; cited 2010 Jan 21]

[B21] Brooks R EuroQol: the current state of play Health Policy 1996 37 53 72 10.1016/0168-8510(96)00822-6 10158943

[B22] The EuroQol Group Valuation of EQ-5D: Value Sets http://www.euroqol.org/eq-5d/valuation-of-eq-5d/value-sets.html (accessed 26 July 2010)

[B23] Yesavage J Brink T Development and validation of a geriatric depression screening scale: a preliminary report J Psychiatr Res 1983 17 37 49 10.1016/0022-3956(82)90033-4 7183759

[B24] Kørner A Lauritzen L Abelskov K Gulmann N Brodersen A Wederwang-Jensen T Kjeldgaard K The Geriatric Depression Scale and the Cornell Scale for Depression in Dementia. A validity study Nord J Psychiatry 2006 60 360 364 10.1080/08039480600937066 17050293

[B25] Stek M Vinkers D Gussekloo J van der Mast R Beekman A Westendorp R Natural history of depression in the oldest old Br J Psychiatry 2006 188 65 69 10.1192/bjp.188.1.65 16388072

